# Progesterone as a Postnatal Prophylactic Agent for Encephalopathy Caused by Prenatal Hypoxic Ischemic Insult

**DOI:** 10.1210/en.2018-00148

**Published:** 2018-04-10

**Authors:** Yoshimasa Kawarai, Hirokazu Tanaka, Tatsuya Kobayashi, Makio Shozu

**Affiliations:** 1 Department of Reproductive Medicine, Graduate School of Medicine, Chiba University, Chiba, Japan; 2 Department of Obstetrics and Gynecology, School of Medicine, International University of Health and Welfare, Narita, Japan

## Abstract

Brain damage caused by hypoxic ischemic insult during the perinatal period causes hypoxic ischemic encephalopathies (HIEs). Therapeutic hypothermia is indicated for HIE, but because the therapeutic burden is large for its limited therapeutic effectiveness, another strategy is needed. Progesterone (P_4_) plays a neuroprotective role through the actions of its metabolite, allopregnanolone (Allo), on P_4_ receptor, *γ*-aminobutyric acid type A receptors or both. We examined the therapeutic potential of P_4_ using a newborn rat model of HIE. Fetal rats were exposed to transient ischemic hypoxia by 30-minute bilateral uterine artery clamping on gestational day 18. After spontaneous birth, newborn pups were subcutaneously injected with P_4_ (0.10 or 0.01 mg), medroxyprogesterone acetate (MPA; 0.12 mg), or Allo (0.10 mg) through postnatal days (PDs) 1 to 9. Brain damage in the rats was assessed using the rotarod test at PD50. The HIE insult reduced the rats’ ability in the rotarod task, which was completely reversed by P_4_ and Allo, but not by MPA. Histological examination revealed that the HIE insult decreased neuronal (the cortex and the hippocampal CA1 region) and oligodendroglial cell density (the corpus callosum) through PD0 to PD50. The axon fiber density and myelin sheath thickness in the corpus callosum were also reduced at PD50. The time-course study revealed that P_4_ restored oligodendroglial cells by PD5, which was followed by neuroprotective action of P_4_ that lasted long over the injection period. These results suggest that P_4_ protects the neonatal brain from HIE insult via restoration of oligodendroglial cells.

## Teaser

Progesterone injection in neonatal rats protects against encephalopathy caused by transient ligation of maternal uterine arteries *in utero*.

Hypoxic insults during the perinatal period cause neonatal hypoxic ischemic encephalopathy (HIE), which leads to secondary injuries, such as reperfusion injury, edema, increased intracranial pressure, impaired autoregulation, and hemorrhage, which are important pathologies of later neurodevelopmental impairments, such as epilepsy, behavioral deficits, learning disorders, and cerebral palsy (CP). Recent accumulating evidences suggest that HIE is also associated with autism and attention-deficit/hyperactivity disorder (1).

The occurrence of CP is strongly associated with gestational age at birth: CP is 40 times more prevalent in preterm infants who are born before 28 weeks of gestation than in term infants (2). By contrast, term infants with CP have no history of causative asphyxiating event in over 90% of the cases. However, it is supposed that some hypoxic insults occur *in utero*, but are not identified later, because the term infants often experience hypoxia-related symptoms, such as fetal growth restriction and/or chronic or acute placental pathologies.

Currently, therapeutic hypothermia is indicated for a high-risk neonate to prevent the occurrence of HIE. Hypothermia reduces the death or moderate-to-severe disability rate from 62% to 44% (3). This implies that, even with hypothermia, ~40% of the affected neonates are left disabled. There are other options, such as melatonin, *N*-acetylcysteine, and magnesium sulfate, but their therapeutic effect has not been established (4). A clinical trial revealed that autologous umbilical cord blood transfusion for HIE is feasible, but the therapeutic effect remains to be determined (5, 6).

During pregnancy, a large amount of progesterone (P_4_) is increasingly produced by the placenta and secreted into the maternal plasma, leading to a level as high as 200 ng/mL at term. P_4_ plays important roles in maintaining uterine quiescence through many pathways. For example, P_4_ increases the expression of zinc finger E-box binding homeobox proteins 1 and 2, which suppresses the expression of oxytocin receptors and connexin 43 (7–9). In addition to the maternal plasma, 15% of the P_4_ produced in the placenta enters the fetal plasma, leading to levels as high as 400 to 600 ng/mL, which is higher than that in the mother. However, the role played by such a high level of P_4_ has not yet been determined (10, 11).

In the brain, P_4_ is synthesized in neurons, and metabolized to active neurosteroids, some of which play neuroprotective roles through the *γ*-amino butyric acid type A (GABA_A_) receptor. In animal experiments, P_4_ administration has been shown to improve various neurologic diseases, including traumatic brain injury (TBI), ischemia, spinal cord injury, peripheral nerve injury, demyelinating diseases, neuromuscular disorders, and seizures (12, 13). Therefore, it might be reasonable to suppose that the high level of fetal P_4_ enters the fetal brain, plays a favorable role in fetal brain development, and protects against HIE insult *in utero*. Moreover, preterm birth might deprive the neonate of P_4_’s benefit, and increase susceptibility to HIE.

In this study, we examined whether P_4_ protects the neonatal brain from preterm HIE insult. We used a newborn rat model with preterm HIE insult and postnatal P_4_ treatment, considering the practical therapeutic window to be the postnatal day (PD) after birth.

## Materials and Methods

### Animals

All animal procedures were approved by the Institutional Animal Care and Use Committee of the Chiba University Graduate School of Medicine. Nulliparous Wistar rats (CLEA Japan, Inc., Tokyo, Japan) aged 10 to 18 weeks, were maintained at the same center with *ad libitum* access to food and water. They were individually housed in acrylic cages under a 12-hour light: 12-hour dark cycle, and controlled temperature (24.5°C ± 2°C) and humidity (50% ± 10%).

We used rats that survived hypoxic ischemic insult in our experiments, regardless of sex. We included rats of both sexes for the analysis, because we did not find significant differences, based on sex, in the survival rate, histological findings, and rotarod scores, although the body weight of female rats was lower than that of male rats (Supplemental Fig. 1). There is no sex-related difference in the plasma level of P_4_ through the prenatal days and PDs (14). For histological examination, only male rats were used. A total of 344 rats (158 males and 186 females) were included.

### Rat HIE model

We used rat pups with HIE induced by transient bilateral uterine artery clipping during pregnancy [modified bilateral uterine artery ligation (BUAL)] (15–17). On gestational day 18, pregnant rats were anesthetized using ketamine (75 mg/kg) (Ketalar; Daiichi Sankyo Co., Ltd., Tokyo, Japan) and xylazine (10 mg/kg) (Selactar; Byer Yakuhin, Ltd., Osaka, Japan) via intraperitoneal injection. A midline laparotomy was performed, and the uterine horns were externalized. The four uterine arteries were exposed and pinched with aneurysm clips (KN-353; Natsume Seisakusho Co., Ltd., Tokyo, Japan). For sham groups, the uterine horns were externalized without clipping. After 30 minutes, the clips were removed, and the uterine horns were replaced in the abdomen. Dams were awakened from anesthesia, monitored to ensure adequate recovery, and returned to the animal facility. Pups spontaneously born around gestational day 22 were included in the neonatal HIE models.

### Drugs

We dissolved P_4_ in sesame oil (Progehormon; Mochida Pharmaceutical Co., Ltd, Tokyo, Japan), and diluted it with sesame oil (Sigma-Aldrich, St. Louis, MO) to final concentrations of 1.0 and 0.1 mg/mL. Medroxyprogesterone acetate (MPA) (Sigma-Aldrich) and 3*α*,5*α*-tetrahydroprogesterone [allopregnanolone (Allo)] (Tocris Bioscience, Bristol, United Kingdom) were dissolved in chloroform and mixed with sesame oil to final concentrations of 1.2 and 1.0 mg/mL, respectively. The mixtures were heated at 65°C for 30 minutes to achieve complete evaporation of chloroform.

From PD1 to PD9, animals subcutaneously received prepared drug doses once a day (*i.e.,* P_4_, 0.10 mg/d; MPA, 0.12 mg/d; Allo, 0.10 mg/d).

### Rotarod test

At PD50, a rotarod test (Rat Rota-Rod NG; Ugo Basile, Monvalle, Italy) was performed to assess neurobehavioral function. Before a performance assessment, we trained rats once per day for 5 consecutive days. Each rat was placed individually on a rod rotating at 2 rpm for 5 minutes. For the measurement, after habituation for 3 minutes, the rotarod test was started at 2 rpm, with the speed increasing stepwise every 30 seconds. After 300 seconds, the speed reached 20 rpm. We recorded the latency to falling off the rod (latency-to-fall off). If a rat remained on the rod at 300 seconds, we aborted the test at 300 seconds (18).

### Histological analysis

Rats were euthanized at PD0 to PD50 for histological analysis. The rats were deeply anesthetized with sodium pentobarbital (Kyoritsu Seiyaku Co., Tokyo, Japan), and received intracardiac perfusion of phosphate-buffered saline followed by 4% buffered paraformaldehyde. Then the brain was removed and immersed in 4% buffered paraformaldehyde for at least 2 days before histological processing. They were cut into segments using a rodent brain matrix, and then the segment containing the rostral edge of the third ventricle and the hippocampus was embedded in paraffin wax. Five-micrometer sections were prepared at ~0.4 mm [for the corpus callosum (CC) and the cortex] and 3.3 mm (for the hippocampus) caudal to the bregma, using a sledge microtome. Then they were subjected to histological analyses or immunohistochemistry.

### Immunohistochemistry and morphometry analyses

Deparaffined sections (5-µm thickness) were subjected to immunohistochemistry as previously described (19). Briefly, they were washed with 0.1% Triton X-100 (Sigma-Aldrich) in Tris-buffered saline, and incubated for 30 minutes with one of the following antibodies: mouse anti-NeuN [Research Resource Identifier (RRID): AB_2298772; 1:200; Merck Millipore, Darmstadt, Germany], rabbit anti-Olig2 (RRID: AB_570666; 1:500; Merck Millipore), or rabbit anti-Iba1 (RRID: AB_2665520; 1:500; Wako Pure Chemical Industries, Ltd., Osaka, Japan). Sections were then washed thoroughly with Tris-buffered saline, and incubated with anti-rabbit (RRID: AB_2336810; Vector Laboratories, Burlingame, CA) or anti-mouse immunoglobulin G conjugated to horseradish peroxidase (RRID: AB_2313581, Vector Laboratories) for 30 minutes. Negative controls were subjected to the same procedure without the primary antibody.

Cell counting was performed by a blinded investigator. Immunoreactive cells were manually counted under ×200 magnification. Four adjacent, nonoverlapping square fields (100 × 100 µm) were sampled.

### Transmission electron microscopy

Tissue samples for transmission electron microscopy were fixed in phosphate-buffered 2% glutaraldehyde, and subsequently postfixed in 2% osmium tetroxide for 3 hours in an ice bath. The specimens were then dehydrated in graded ethanol, and embedded in epoxy resin. Ultrathin sections were obtained using an ultramicrotome, stained with uranyl acetate for 10 minutes and modified Sato lead solution for 5 minutes, and submitted for transmission electron microscopy observation (JEM-1200EX; JEOL Ltd., Tokyo, Japan) (20). Quantifications were performed on five images per rat (at least 100 axons per rat).

### Statistical analysis

Kaplan-Meier analysis was used to estimate the latency-to-fall off measured by the rotarod test. Latency-to-fall off was censored at >300 seconds. Comparisons of estimated measures from the rotarod test between the two groups were performed using a two-sided log-rank test. Comparisons of morphometric measures were performed between the two groups using the Wilcoxon rank-sum test, and among the three groups using the Steel-Dwass multiple comparisons test because of small sample sizes. The software JMP 11.2 (SAS Institute, Inc., Cary, NC) was used to perform all statistical analyses. Statistical significance was defined as *P* < 0.05.

## Results

### P_4_ and Allo, but not MPA, reverse motor coordination impairment in HIE

**Figure 1.**
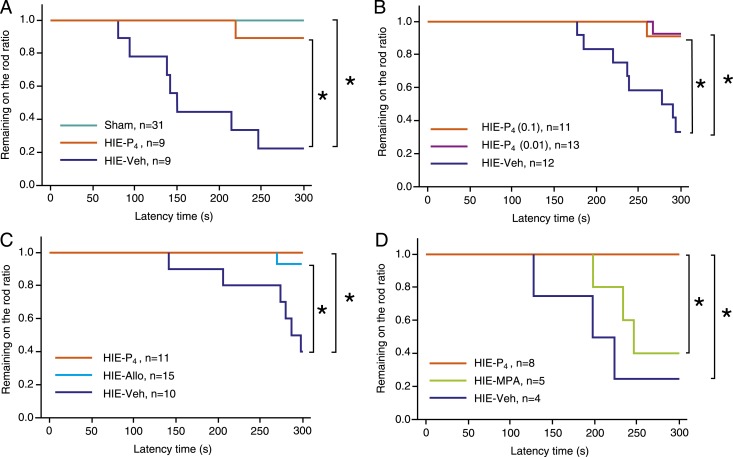
Rotarod test results at PD50, analyzed using the Kaplan-Meier method. We defined the time to remaining on the rotating rod as the latency-to-fall off. The maximum trial length was 300 seconds. (A) Sham vs HIE-P_4_ vs HIE-Veh; (B) HIE-P_4_ (0.1) vs HIE-P_4_ (0.01) vs HIE-Veh; (C) HIE-P_4_ vs HIE-Allo vs HIE-Veh; (D) HIE-P_4_ vs HIE-MPA vs HIE-Veh. The numbers of female (male) aminol used were as follows: (A) 23 (8), 5 (4), and 4 (5) for Sham, HIE-P_4_, and HIE-Veh; (B) 5 (6), 7 (6), and 7 (5) for HIE-P_4_ (0.1), HIE-P_4_ (0.01), and HIE-Veh; (C) 8 (3), 7 (8), and 5 (5) for HIE-P_4_, HIE-Allo, and HIE-Veh; (D) 4 (4), 3 (2), and 3 (1) for HIE-P_4_, HIE-MPA, and HIE-Veh. *Denotes a significant difference in the latency-to-fall off (*P* < 0.05) detected using the Kaplan-Meier method. HIE-Allo, allopregnanolone-treated hypoxic ischemic encephalopathy group; HIE-MPA, medroxyprogesterone acetate-treated hypoxic ischemic encephalopathy group; HIE-P_4_, progesterone-treated hypoxic ischemic encephalopathy group; HIE-P_4_ (0.01), 0.01-mg/d progesterone-treated hypoxic ischemic encephalopathy group; HIE-P_4_ (0.1), 0.1-mg/d progesterone-treated hypoxic ischemic encephalopathy group; HIE-Veh, vehicle-treated hypoxic ischemic encephalopathy group; Sham, sham-operated group.

We measured motor coordination at PD50 using the rotarod test, and analyzed the latency to falling off (latency-to-fall off). The preterm HIE insult significantly shortened the latency-to-fall off (vehicle-treated HIE group vs sham group, *P* < 0.05, log-rank test; Fig. 1A). The P_4_ treatment restored the latency-to-fall off in the vehicle-treated HIE group to the level of the sham group. The lower dose of P_4_ (0.01 mg/d) restored the latency-to-fall off similar to the higher dose (0.1 mg/d) (lower dose P_4_-treated group vs higher dose P4-treated group, *P* = 0.88; Fig. 1B), and the difference between the vehicle-treated HIE group and the low dose P_4_-treated groups was significant (*P* < 0.05, log-rank test).

The effect of P_4_-related compounds was examined; Allo, a metabolite of P_4_, restored the latency-to-fall off in the HIE model as P_4_ did (Allo-treated HIE group vs P_4_-treated HIE group, *P* = 0.39; Allo-treated group vs vehicle-treated HIE group, *P* < 0.05; Fig. 1C). In contrast, MPA, a synthetic agonist for the classic intracellular P_4_ receptor (PR), did not restore the latency-to-fall off (MPA-treated HIE group vs vehicle-treated HIE group, *P* = 0.35; MPA-treated group vs P4-treated group, *P* < 0.05; Fig. 1D). We conducted the same experiment without acclimation to the rotarod, and confirmed that P4, but not MPA, restored the latency-to-fall off of HIE rats (Supplemental Fig. 1D). Again, P_4_ treatment was similarly effective in male and female rats (Supplemental Fig. 1E–1H).

### Histological changes in neurons and oligodendroglial cells

Histological changes caused by HIE injury, and restoration by P_4_, were examined using immunohistochemistry on PD50. The numbers of immunoreactive cells in the cortex or the CA1 region of the hippocampus were counted in four different square fields (0.01 mm^2^ each) for each animal.

**Figure 2.**
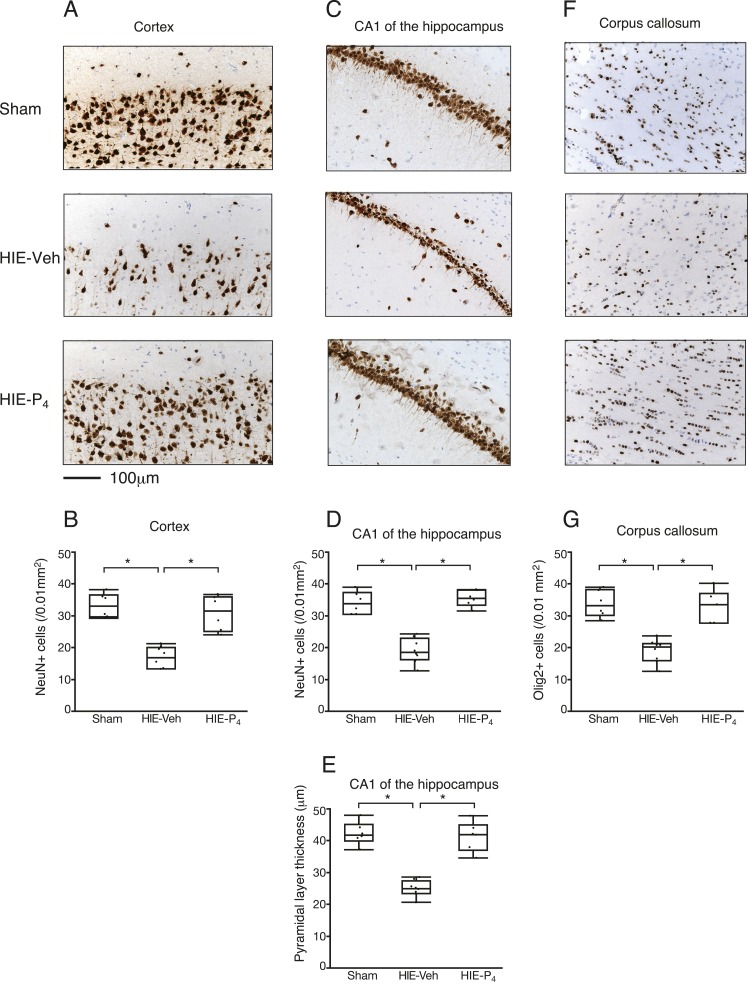
Immunohistochemistry of the neurons and oligodendrocytes at PD50. (A) Representative images of the cortex stained with anti-NeuN antibodies. (B) The number of NeuN-positive cells in the cortex. (C) Representative images of the CA1 region of the hippocampus stained with anti-NeuN antibodies. (D) The number of NeuN-positive cells in the CA1 region of the hippocampus. (E) Thickness of the CA1 pyramidal layer. (F) Representative images of the CC stained with anti-Olig2 antibodies. (G) The number of Olig2-positive cells in the CC. The body of the box plot represents the distribution’s interquartile range and median. The whiskers extend from the quartiles to the last data point within 150% of the interquartile range, with the outliers beyond represented as dots. The number of animals used for morphometry were 6, 8, and 6 for Sham, HIE-Veh, and HIE-P_4_, respectively. Scale bars = 100 µm. *Denotes a significant difference (*P* < 0.05) detected by the Steel-Dwass multiple comparisons test. The powers of the analysis are 1.00 for all three measures. HIE-P_4_, 0.1-mg/d progesterone-treated hypoxic ischemic encephalopathy group; HIE-Veh, vehicle-treated hypoxic ischemic encephalopathy group; Sham, sham-operated group.

In both the cortex and CA1, the number of NeuN-positive cells was higher in the P_4_-treated HIE group than in the vehicle-treated HIE group (*P* < 0.05, the Steel-Dwass multiple comparisons test), and was equivalent to that in the sham group (Fig. 2A–2D). Moreover, P_4_ thickened the CA1 pyramidal layer to the level of the sham group (*P* < 0.05) (Fig. 2E).

Oligodendroglial cells, which are responsible for myelination, were assessed using an anti-Olig2 antibody. In the CC, the number of Olig2-positive cells increased more in the P_4_-treated HIE group than in the vehicle-treated HIE group (*P* < 0.05), and was equivalent to that in the sham group (Fig. 2F and 2G). Moreover, P_4_ accelerated a linear arrangement of oligodendroglial cells along the axons, as observed in the sham group (Fig. 2F).

### Effect of P_4_ on HIE-induced microglial activation

**Figure 3.**
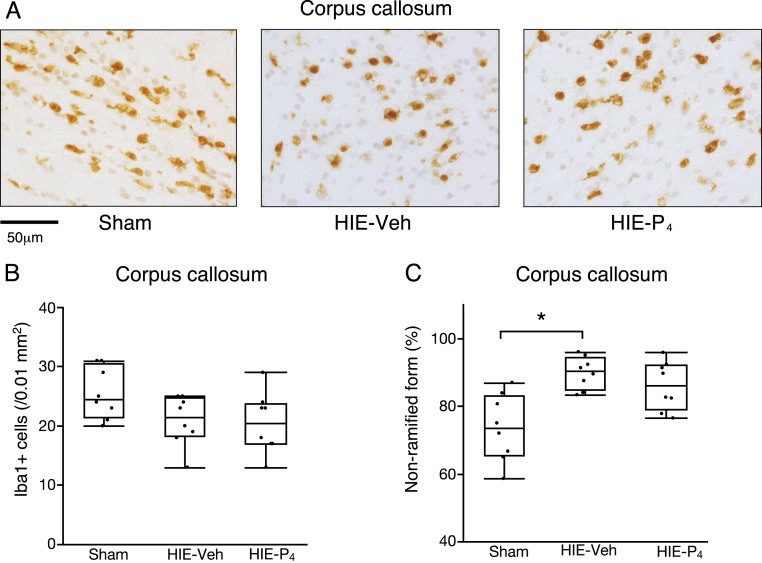
Immunohistochemistry of the microglia at PD9. The number and morphology of the CC microglia are shown. (A) Representative images of the CC stained with the anti-Iba1 antibody. Scale bar = 50 µm. (B) The number of Iba1-positive cells. (C) The percentage of CC microglia in the nonramified (activated) form. The numbers of animals used were 8 per group. *Denotes a significant difference (*P* < 0.05) detected by the Steel-Dwass multiple comparisons test. The power of the analysis is 0.31 and 0.93 for Iba1 + cells and nonramified form (%), respectively. HIE-P_4_, 0.1-mg/d progesterone-treated hypoxic ischemic encephalopathy group; HIE-Veh, vehicle-treated hypoxic ischemic encephalopathy group; Sham, sham-operated group.

Microglia, the central nervous system’s resident macrophages, were examined immunohistochemically using the anti-Iba1 antibody (Fig. 3A).

Microglia in the CC were observed on PD9 to examine the direct immunosuppressive action of P_4_. Density of microglia was higher in the sham group than in the vehicle-treated HIE and P4-treated HIE groups, but the differences were not statistically significant (Fig. 3B). The fraction of nonramified forms, representing the activated form of microglia, to the total number of microglia was significantly higher in the vehicle-treated HIE group than in the sham group (*P* < 0.05; Fig. 3C). The fraction in P4-treated HIE group was between those of the vehicle-treated HIE group and the sham group, and the differences with any of the other two groups were not significant.

### P_4_ alters temporal changes in neuronal and oligodendroglial cell density

**Figure 4.**
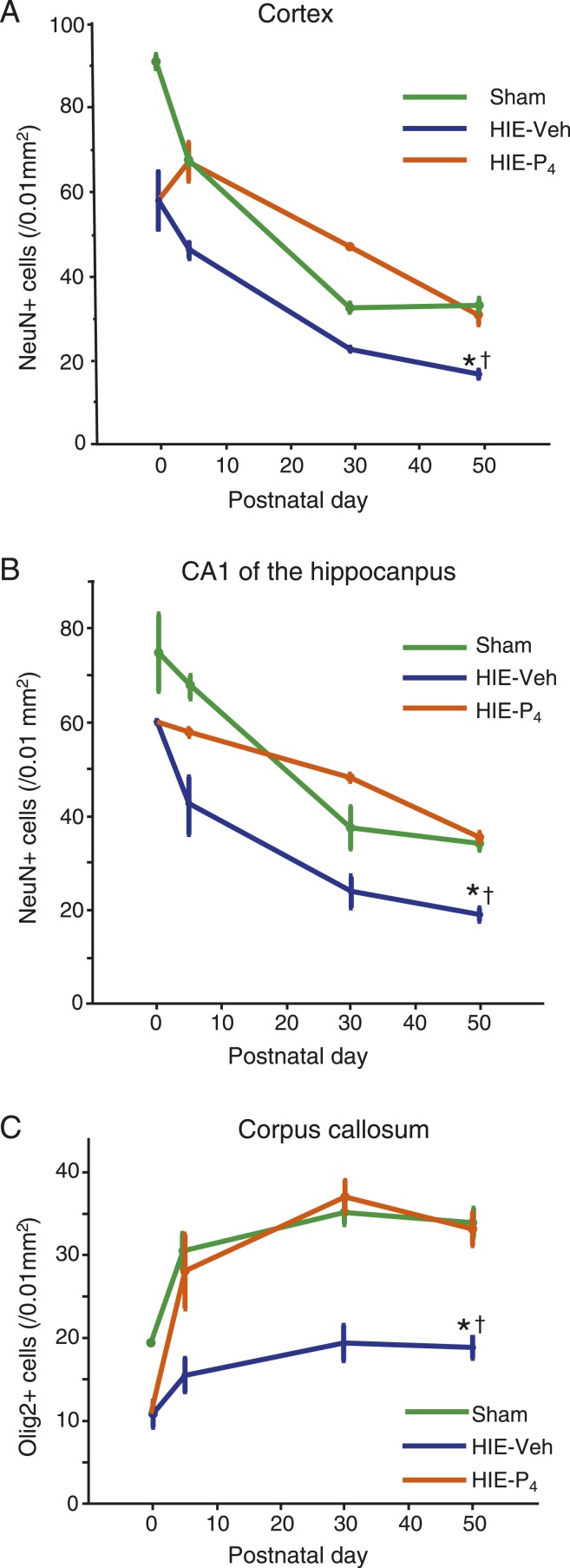
The temporal changes in neuronal and oligodendroglial cell density. (A) NeuN-positive cortical cells. (B) NeuN-positive CA1 cells. (C) Olig2-positive CC cells. Number of animals used for each time-point are as follows: 3 (PD0), 2 (PD5), 2 (PD30), and 8 (PD50) for HIE-P4; 3 (PD0), 2 (PD5), 2 (PD30), and 8 (PD50) for HIE-Veh; and 2 (PD0), 3 (PD5), 3 (PD30), 6 (PD50) for Sham group. *†Denotes a significant difference (*P* < 0.05) detected by the Steel-Dwass multiple comparison tests on PD50: *Sham vs HIE-Veh; †HIE-P4 vs HIE-Veh. The powers of the analysis for PD5, PD30, and PD50 were as follows: 0.96, 1.00, and 1.00 (cortex); 0.85, 0.75, and 1.00 (hippocampus); 0.61, 0.94, and 1.00 (CC). HIE-P4, 0.1-mg/d progesterone-treated hypoxic ischemic encephalopathy group; HIE-Veh, vehicle-treated hypoxic ischemic encephalopathy group; Sham, sham-operated group.

At PD0, PD5, PD30, and PD50, the numbers of NeuN-positive cells in the cortex and CA1, and Olig2-positive cells in the CC were assessed. The vehicle-treated HIE group’s neuronal density was lower than that of the sham group at each PD, but its decay slopes were similar to those of the sham group (Fig. 4A and 4B). On the other hand, the P_4_-treated HIE group had a lower neuronal density in both the cortex and CA1 than the sham group at PD0, but showed a transient increment of neurons in the cortex and shallower decay slope in the CA1 until PD30. Eventually, the P_4_-treated HIE group had the same density as the sham group at PD50 (Fig. 4A and 4B).

With respect to the numbers of oligodendroglial cell, the P_4_-treated HIE group caught up with the sham group by PD5, and maintained the same levels as the shame group thereafter (Fig. 4C).

### P_4_ enhances myelination

**Figure 5.**
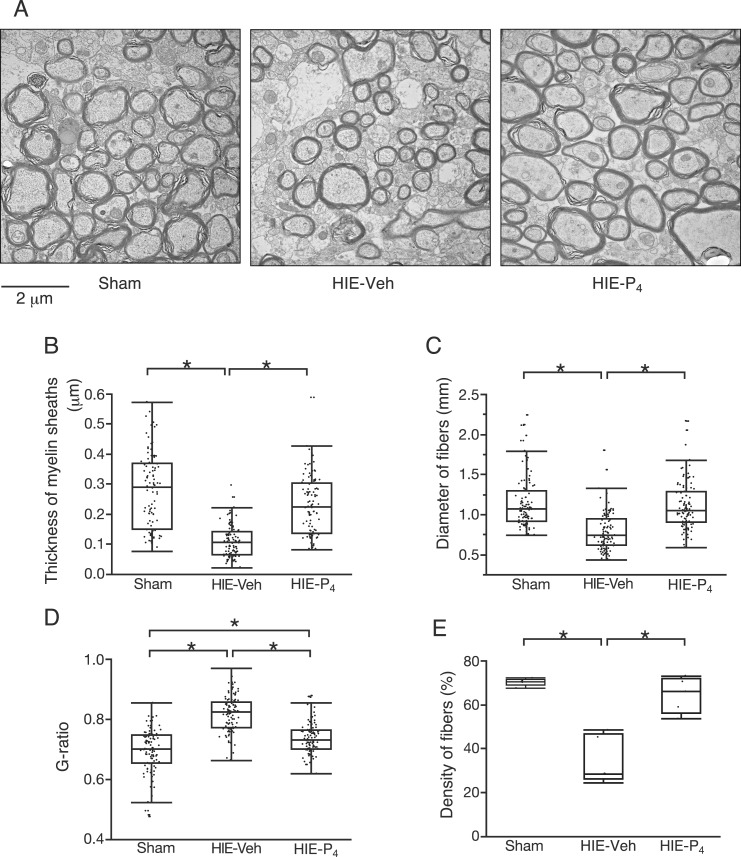
Myelination in the CC. (A) Electron microscopic images of the CC at PD50 (vertical section to the axons). (B) Thickness of the myelin sheaths. (C) Diameter of the neuron fibers. (D) G-ratio (axon diameter/fiber diameter ratio). (E) Density of the neuron fibers. The ratio of the area occupied by neuron fibers to the total area observed. Five rats were used per group. Morphometry of axon fibers was conducted on 20 randomly selected fibers per rat. The results from a total of 100 axons are plotted for each group in (B–D). Scale bar = 2 µm. *Denotes a significant difference (*P* < 0.05) detected by the Steel-Dwass multiple comparison test. HIE-P_4_, 0.1-mg/d progesterone-treated hypoxic ischemic encephalopathy group; HIE-Veh, vehicle-treated hypoxic ischemic encephalopathy group; Sham, sham group.

Using electron microscopy, myelin sheaths in the CC from the vehicle-treated HIE group were observed as thinned black layers around the axon (Fig. 5A). Myelin thickness and axon diameters were measured. In the P_4_-treated HIE group, axons were better myelinated and thicker than those in the vehicle-treated HIE group, and similar to those in the sham group (Fig. 5B–5D). Moreover, the density of nerve fibers (nerve fiber occupying area/total area) was also restored by P_4_ treatment (Fig. 5E).

## Discussion

This study demonstrates that postnatal P_4_ administration reverses neurobehavioral dysfunction caused by prenatal hypoxic ischemic insult. The histological examination provided evidences that P_4_ administration increased the neuronal and oligodendroglial cell density in the developing brain, and enhanced preservation of neural fibers and their myelination, eventually to the levels of control animals.

### P_4_ and Allo improved neurobehavioral function

It has been demonstrated that P_4_ exerts a neuroprotective effect against TBI and stoke-induced brain damage, although the mechanism of action and the involved receptor are yet to be explored (21–23). In this study, both P_4_ and its metabolite, Allo, restored the neurobehavioral dysfunctions, whereas MPA did not. There are several possible explanations for the difference in the therapeutic effectiveness among these three compounds; Allo and the 3*α*,5*α*-tetrahydro metabolites of P_4_ bind to the neurosteroid-binding site of GABA_A_ receptors, whereas the 3α,5α-metabolites of MPA do not bind to the neurosteroid-binding site of GABA_A_ receptors but bind to PRs (24, 25). Moreover, MPA can bind to other steroid and nonsteroid receptors, and may have adverse effects on neural cells (26, 27). Thus, an explanation is that Allo and P_4_ act by binding to the GABA_A_ receptor. However, Allo can be back-oxidized to 5*α*-dihydroprogesterone by 3*α*-hydroxysteroid dehydrogenase, and is able to bind to PR (22). Thus, another explanation is that Allo and P_4_ act by binding to the intracellular PRs, which are widely expressed in the brain. Moreover, new membrane receptors of P_4_, including membrane PR and P_4_ membrane receptor component 1, are also being studied with regard to the neuroprotective action of P_4_ recently (23). The mechanism of action of P_4_ is yet to be determined.

### P_4_ rescues neurons probably through action on oligodendroglial cells

Our time-course study supports the notion that P_4_ repairs oligodendrocyte progenitor cells initially, which then rescue neurons. During the development, the oligodendrocyte density increases, while the neuronal density decreases with advancing age, due to cell death. The HIE procedure that we used decreased both the oligodendroglial cell (Fig. 4C) and neuronal density (Fig. 4B and 4C) by 20% to 50% compared with that of the control at PD0. Interestingly, P_4_ injection restored the oligodendroglial cell density by PD5, after which the density was identical to that of the control (Fig. 4C). By contrast, P_4_ injection attenuated or cancelled the age-dependent decrease in the neuronal density at PD5; subsequently, the attenuating effect lasted until PD30, and was lost by PD50 (Fig. 4A and 4B). The effect of P_4_ on the neuronal density seemed to reach its maximum at around PD30, when compared with the age-matched controls. The initial response of oligodendroglial cells during the P_4_ injection period, followed by prolonged response in neurons beyond the P_4_ injection period, is compatible with the notion that P_4_ primarily acts on oligodendroglial cells to stimulate their generation from progenitor cells, which then protects against neuronal loss.

### P_4_ restores neurobehavioral function through the enhancement of myelination

We speculated that P_4_-induced restoration of myelin and axon preservation is the direct mechanism underlying the recovery of neurobehavioral dysfunction, based on morphological findings. According to the morphological restoration of the oligodendroglial cells, the thickness of myelin sheaths (Fig. 5B), axon diameters, and axon densities increased upon P_4_ injection (Fig. 5B–5D). We conclude that P_4_ restores oligodendrocytes, protects neurons, and in turn, enhances axon preservation and myelin sheath formation, which eventually maintains neuromotor coordination. The same consequence following oligodendrocyte recovery has been reported in the epidermal growth factor-treated preterm brain injury models, in which neuromotor coordination disorder and cellular damage were reversed though oligodendrocyte recovery (28).

Large-diameter axons may be more sensitive to the HIE insult, and the P_4_-induced recovery of the myelin sheath seems to be less sufficient in large-diameter axons than in small-diameter axons. As shown in Fig. 5B, the HIE animals showed reduced number of axons, specifically those with thicker myelin sheaths. Moreover, P_4_ treatment increased the number of axons with thinner myelin sheath efficiently but increased the number of axons with thicker myelin sheath less efficiently (Fig. 5B). The axons with thick sheaths generally have a large diameter, which is required for fast transmission between the sensory-motor and visual regions (29). The diameter distribution of axons in the CC is altered in autism, dyslexia, and even in schizophrenia. A wide distribution of axon diameter is also found following exposure to neurotoxic drugs, such as alcohol. Our results indicate that the larger the diameter of the axon, the more sensitive it is to the HIE insult, and the more difficult is its recovery. Moreover, in terms of distinguishing the effect of P_4_, the density of axon fibers seems to be the best index among the four indices that we examined.

### P_4_ may suppress inflammatory reaction in the brain

Immunosuppression may be involved in the restoration of neurobehavioral function in our experimental model, as a study has shown that P_4_ suppresses the immune system, which is activated by hypoxic ischemic stress-induced inflammation in the brain (30). Microglia is of mesodermal origin; it enters the brain during early development and transform into a branched, ramified form called resting microglia, and resides in the brain. Once some pathological events occur, resident microglia become amoeboid and migrate, proliferate, and phagocytose (31, 32). In our study, the prenatal HIE insult increased activated microglial fractions without significant changes in microglial density, implying that the immune process was activated. Postnatal P_4_ treatment reverted the HIE-induced microglial activation to some extent, abolishing the statistically significant difference with the sham operated group (Fig. 3C). Thus, immune suppression may be involved in the mechanism underlying the restoration of neurobehavioral dysfunction in our model.

### Dose of P_4_

The dose of P_4_ used in this study may be within the physiological range for a human fetus. Both doses (0.1 and 0.01 mg/body) of P_4_ were used to show restoration of neurobehavioral function (Fig. 1A and 1B). The higher dose of 0.1 mg/body roughly corresponds to 20.0 and 5.0 mg·kg^−1^·d^−1^ on PD1 and PD9, respectively. These doses are similar to or somewhat lower than that used for human trials, in which P_4_ (22.5 mg·kg^−1^·d^−1^) and estradiol were supplemented to preterm infants with the intention of reducing mortality and bronchopulmonary dysplasia, as mentioned earlier (33–35). These doses are established to mimic the plasma P_4_ concentration of a preterm human fetus. Moreover, the administration of P_4_ did not affect mortality or the latency-to-fall off in the rotarod test using sham-operated rats, suggesting that the doses of P_4_ are neither toxic nor beneficial (Supplemental Figs. 1–3). Collectively, the dose of P_4_ that we used seem to be feasible for a clinical study, although we must be careful in extrapolating the data to humans, considering the differences between the species.

It is reported that 4 to 8 mg/kg of P_4_ may be more effective for neuroprotection than the higher doses in pubertal rat stroke/hypoxic models (36). In our model, the high dose (20.0 to 5.0 mg·kg^−1^·d^−1^) was as effective as the low dose (2 to 0.5 mg·kg^−1^·d^−1^). This inconsistency in dose-response may be attributed to the difference between the HIE models used (age and types of brain injuries), outcomes measured, and duration of treatment. Administration of gradually decreasing dose in our model makes a head-to-head dose comparison difficult.

### Use of the modified BUAL model and its relevance in humans

The modified BUAL model in rats that we used is one of the reasonable animal models of neonatal HIE (15). Numerous types of animal models have been developed as an HIE model, using various insults, including unilateral common carotid artery occlusion (Vannucci model), bilateral carotid artery occlusion, four-vessel occlusion, middle cerebral occlusion, chronic hypoxia, and cerebrocortical photothrombosis (28, 37–41). Most of these models used permanent occlusions of arteries in the brain. In contrast, the modified BUAL model uses transient interruption of the uterine flow, which mimics a transient, and thus, reversible decrease in the placental blood flow. Therefore, the modified BUAL model seems the most relevant model to humans with neonatal brain injuries associated with perinatal systemic hypoxic insults. Moreover, the brain pathologies observed in the modified BUAL model (oligodendrocyte loss, gliosis, and axonal disruption) also mimics pathologies often observed in humans with neonatal HIE (oligodendrocyte loss, hypomyelination, and astrogliosis) (15, 42). Moreover, full-term rat pups are similar to human preterm infants with respect to developmental features of the brain, including neurulation, proliferation, migration, organization, and myelination (43). Further, the degree of damage can be controlled by the duration of occlusion in the BUAL model so that animals having an intended degree of perturbation can survive until adulthood, and be used for experiments. Thus, this approach is suitable for making neonatal HIE models with various severities of neuropsychological deficits to screen putative neuroprotective compounds (44). In fact, the 30-minute occlusion in our BUAL model almost halved the live birth rate compared with the sham group (Supplemental Fig. 2A), and reduced the birth weights by ~15% compared with the sham group (Supplemental Fig. 2B). Eventually, the 30-minute BUAL attenuated neuronal and oligodendroglial cell density, and impaired neurobehavioral function in rats by PD50. Our model, in which 50% of rats died and survivors sustained neurobehavioral impairment, seems to correspond to mild or moderate HIE in humans, where the surviving children are at high risk for CP, cognitive dysfunction, and epilepsy.

We used the CC to examine the axonal changes. Axons in the CC are formed before the birth, and subsequently, a significant number of the axons are eliminated rapidly after birth in the rat (45, 46). This elimination lasts until PD6, and in turn, axon myelination starts at PD11. Thus, our experimental model is suitable to assess axon fiber preservation.

### P_4_ as a neuroprotective agent for the perinatal brain

We tested the effect of P_4_ because it is a natural compound existing abundantly throughout the fetal life, which suggest the physiological roles of P_4_ and the safety in using it for possible clinical use.

The neuroprotective action observed in this study supports the prophylactic use of P_4_ for neonates who have experienced a hypoxic ischemic insult, and thus, are at a high risk for HIE. Since the 1990s, a large number of experimental studies using animal models revealed the neuroprotective effectiveness of P_4_, which includes protection from cortical contusion injury or TBI (47–51). Moreover, the P_4_ level is elevated in the brain early after contusion in experimental rodents (52). Similarly, the Allo level is increased in the fetal brain after umbilical cord occlusion-induced hypoxic ischemic stress in sheep (53). Although numerous clinical trials have evaluated the neuroprotective effects of P_4_, no clinical benefits have been observed for TBI or stroke thus far (54, 55). Nevertheless, a clinical trial that used estradiol and P_4_ supplementation showed potential benefits for long-term neurodevelopment in infants with extremely low birth weight (56). Supplemental P_4_, as well as estradiol, administered for at most 4 weeks, mimics the blood levels of a fetus at a corresponding age, improves cognitive function, and reduces occurrences of CP in preterm infants (33, 56). The current study provided experimental evidence supporting the findings of human trials. Considering that P_4_ is a natural and the most abundant steroid in fetuses, we speculate that the high level of intrinsic P_4_ plays a neuroprotective role in fetuses.

### Limitation

We conducted the experiments using only two doses of P_4_. More precise titration is needed to identify the lowest dose required to achieve the maximal effects. Hirst *et al.* (57) reported that some of the P_4_ is converted to cortisol, a hormone that could be detrimental to brain development. Thus, we need to consider the combined effects of possible P_4_ metabolites as well as the diverse effects of steroids. We assessed inflammatory response only at PD9, and only using immunohistochemistry. Before drawing conclusions regarding the contribution of inflammatory responses, it is necessary to repeat the experiments at multiple time-points after P_4_ treatment, and by using complementary and qualitative methods, such as western blotting.

We did not detect sex-related difference in the effect of P_4_, although there are several reports on the sex-related differences in the recovery from hypoxic brain injury (58). This might be because the number of animals was too low to detect the sex-related difference, although sufficient to detect the effect of treatment when the data from both sexes were combined.

## Conclusions

This study shows that early P_4_ administration reverses neonatal HIE, probably through the GABA_A_ receptor signaling of oligodendroglial cells. This study suggests that P_4_ injection could be a preventive agent against perinatal HIE insults, which might be relevant to CP and other cognitive disorders (1, 59).

## Supplementary Material

Supplemental Figures

## Abbreviations:

Allo: allopregnanolone
BUAL: bilateral uterine artery ligation
CC: corpus callosum
CP: cerebral palsy
GABA_A_: *γ*-amino butyric acid type A
HIE: hypoxic ischemic encephalopathy
MPA: medroxyprogesterone acetate
P_4_: progesterone
PD: postnatal day
PR: progesterone receptor
RRID: Research Resource Identifier
TBI: traumatic brain injury
