# Supplementary material for: Progesterone as a Postnatal Prophylactic Agent for Encephalopathy Caused by Prenatal Hypoxic Ischemic Insult
Source: Endocrinology. 2018 Apr 10;159(6):2264–74. doi: 10.1210/en.2018-00148 (PMC5946846; doi:10.1210/en.2018-00148)
Supplement: Supplemental Figures [file en.2018-00148.sf1.pptx]

## Slide 1
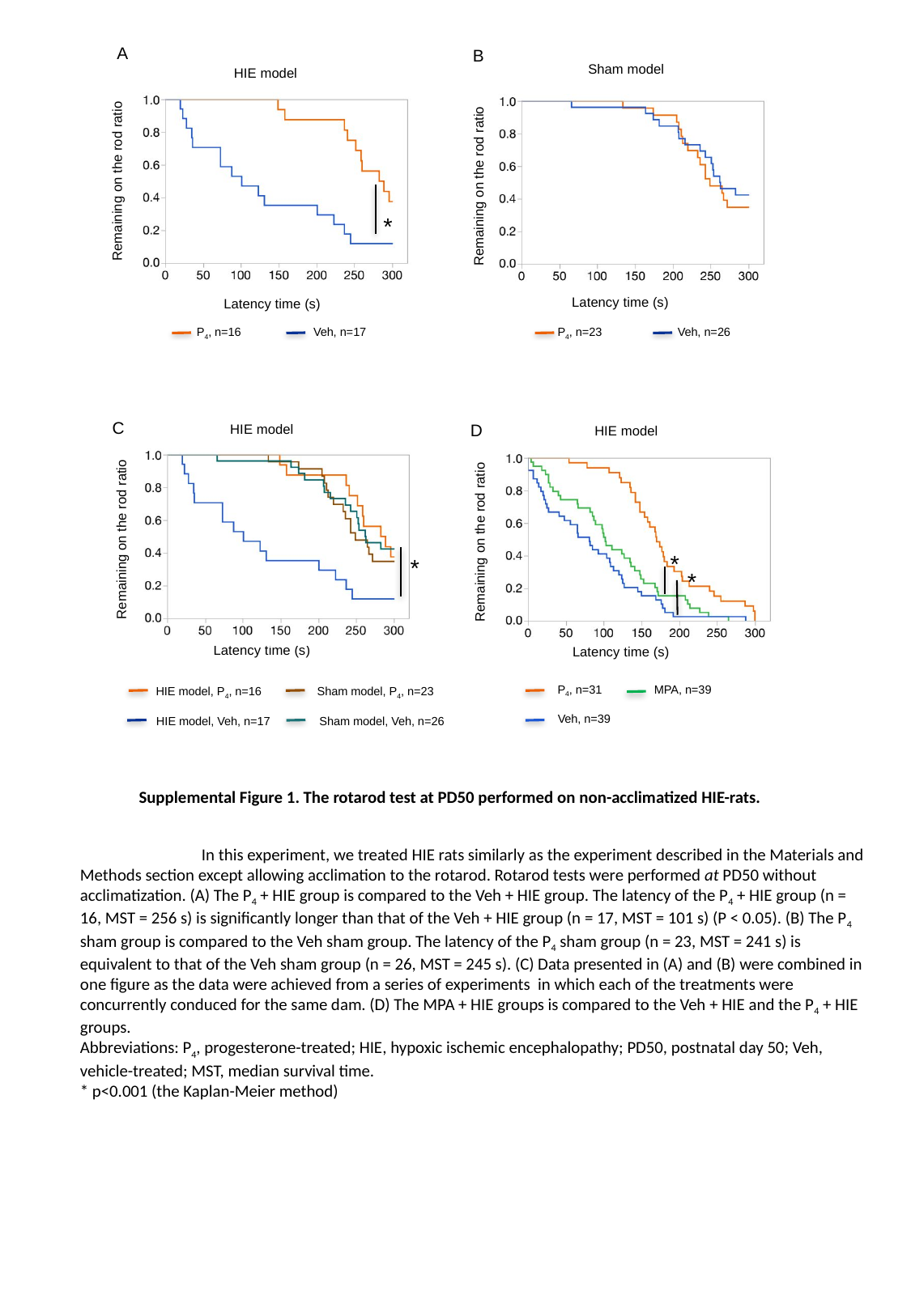

A
B
Sham model
HIE model
Remaining on the rod ratio
Remaining on the rod ratio
*
Latency time (s)
Latency time (s)
P4, n=16
Veh, n=17
P4, n=23
Veh, n=26
C
D
HIE model
HIE model
Remaining on the rod ratio
Remaining on the rod ratio
*
*
*
Latency time (s)
Latency time (s)
P4, n=31
MPA, n=39
HIE model, P4, n=16
Sham model, P4, n=23
Veh, n=39
HIE model, Veh, n=17
Sham model, Veh, n=26
Supplemental Figure 1. The rotarod test at PD50 performed on non-acclimatized HIE-rats.
	In this experiment, we treated HIE rats similarly as the experiment described in the Materials and Methods section except allowing acclimation to the rotarod. Rotarod tests were performed at PD50 without acclimatization. (A) The P4 + HIE group is compared to the Veh + HIE group. The latency of the P4 + HIE group (n = 16, MST = 256 s) is significantly longer than that of the Veh + HIE group (n = 17, MST = 101 s) (P < 0.05). (B) The P4 sham group is compared to the Veh sham group. The latency of the P4 sham group (n = 23, MST = 241 s) is equivalent to that of the Veh sham group (n = 26, MST = 245 s). (C) Data presented in (A) and (B) were combined in one figure as the data were achieved from a series of experiments in which each of the treatments were concurrently conduced for the same dam. (D) The MPA + HIE groups is compared to the Veh + HIE and the P4 + HIE groups.
Abbreviations: P4, progesterone-treated; HIE, hypoxic ischemic encephalopathy; PD50, postnatal day 50; Veh, vehicle-treated; MST, median survival time.
* p<0.001 (the Kaplan-Meier method)

## Slide 2
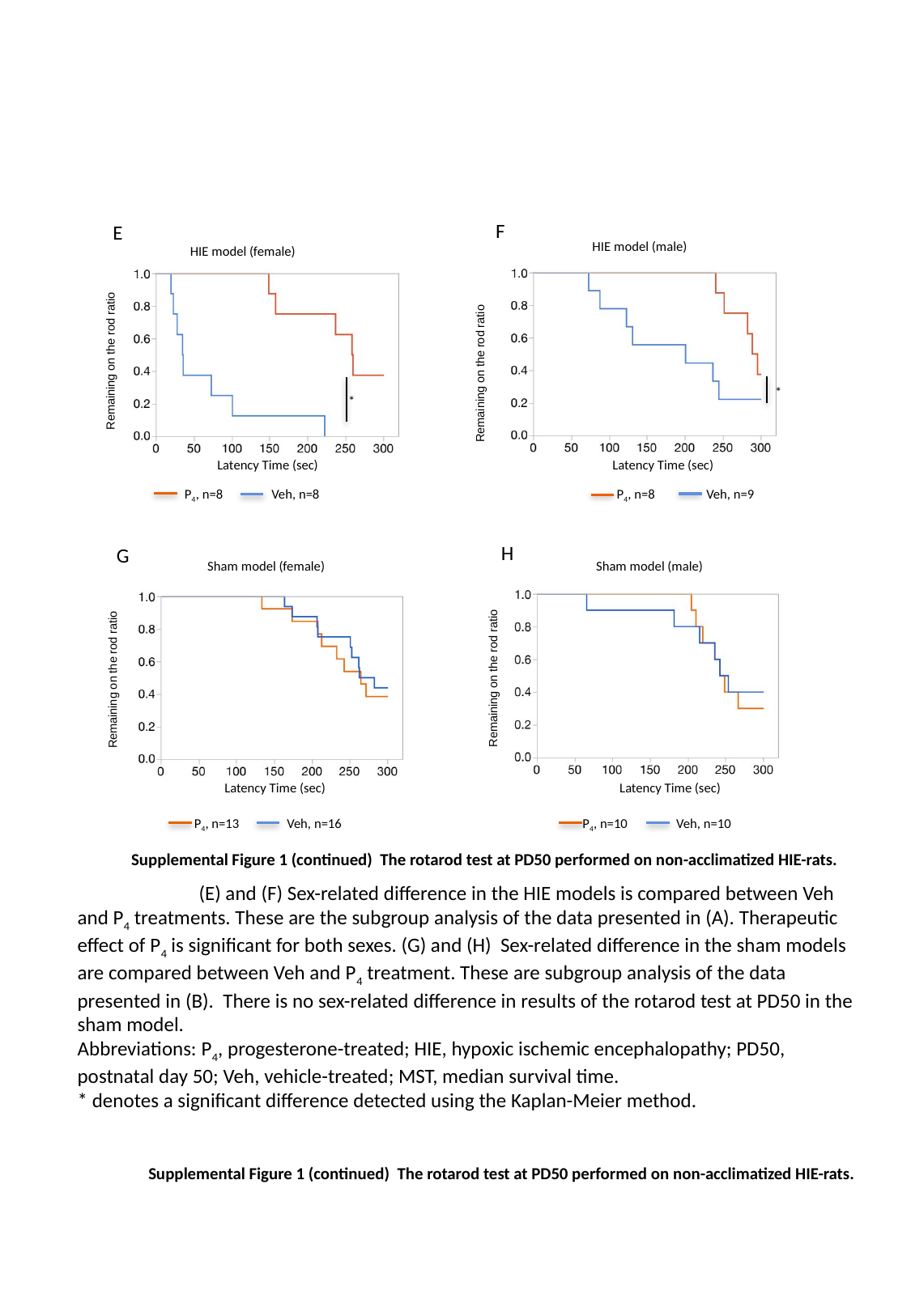

F
E
HIE model (male)
HIE model (female)
Remaining on the rod ratio
Remaining on the rod ratio
*
*
Latency Time (sec)
Latency Time (sec)
P4, n=8
Veh, n=8
P4, n=8
Veh, n=9
H
G
Sham model (female)
Sham model (male)
Remaining on the rod ratio
Remaining on the rod ratio
Latency Time (sec)
Latency Time (sec)
P4, n=13
Veh, n=16
P4, n=10
Veh, n=10
Supplemental Figure 1 (continued) The rotarod test at PD50 performed on non-acclimatized HIE-rats.
	(E) and (F) Sex-related difference in the HIE models is compared between Veh and P4 treatments. These are the subgroup analysis of the data presented in (A). Therapeutic effect of P4 is significant for both sexes. (G) and (H) Sex-related difference in the sham models are compared between Veh and P4 treatment. These are subgroup analysis of the data presented in (B). There is no sex-related difference in results of the rotarod test at PD50 in the sham model.
Abbreviations: P4, progesterone-treated; HIE, hypoxic ischemic encephalopathy; PD50, postnatal day 50; Veh, vehicle-treated; MST, median survival time.
* denotes a significant difference detected using the Kaplan-Meier method.
Supplemental Figure 1 (continued) The rotarod test at PD50 performed on non-acclimatized HIE-rats.

## Slide 3
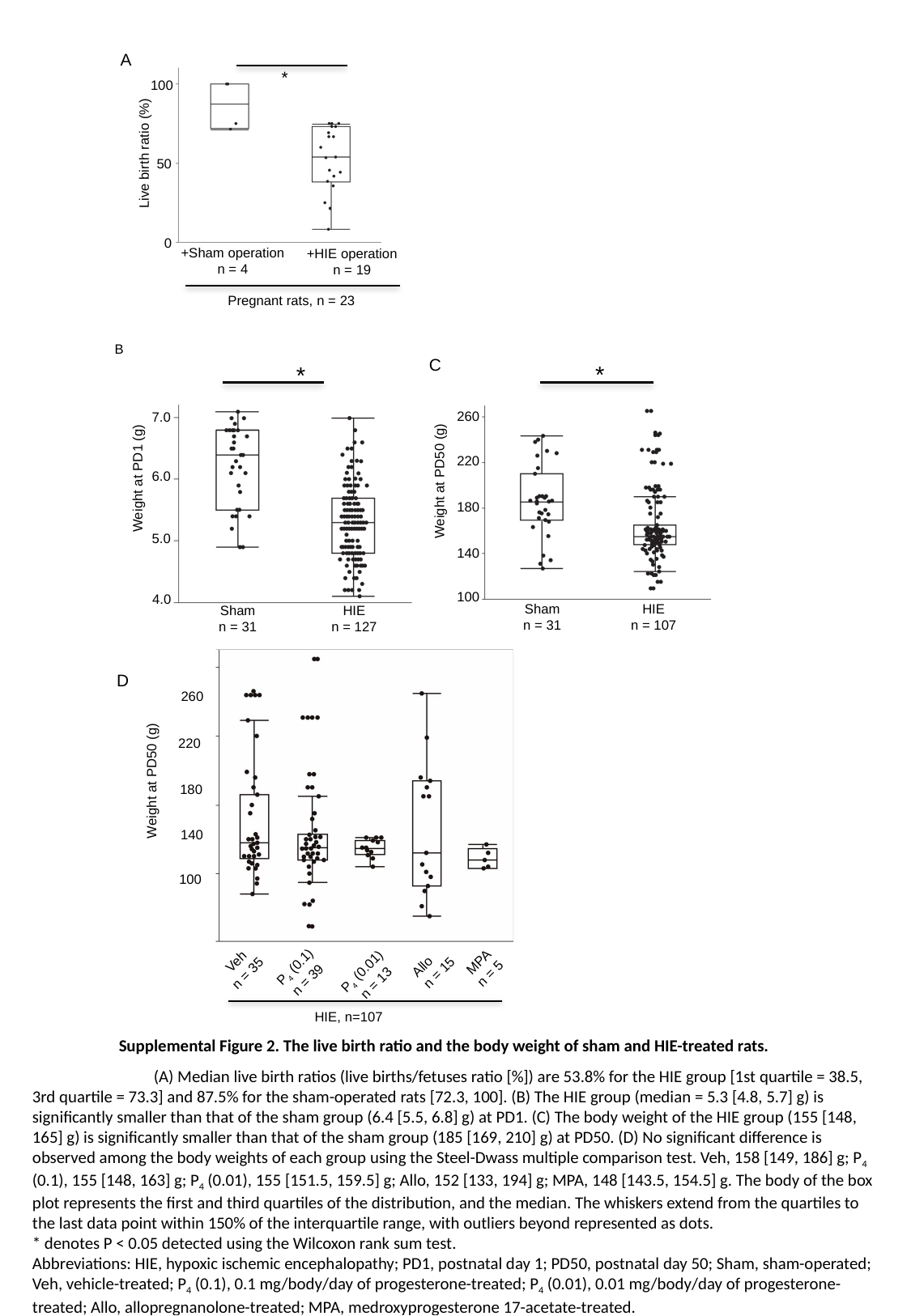

A
*
100
Live birth ratio (%)
50
0
+Sham operation
n = 4
+HIE operation
n = 19
Pregnant rats, n = 23
B
C
*
260
220
Weight at PD50 (g)
180
140
100
Sham
n = 31
HIE
n = 107
*
7.0
Weight at PD1 (g)
6.0
5.0
4.0
Sham
n = 31
HIE
n = 127
260
220
Weight at PD50 (g)
180
140
100
Allo
n = 15
Veh
n = 35
MPA
n = 5
P4 (0.1)
n = 39
P4 (0.01)
n = 13
HIE, n=107
D
Supplemental Figure 2. The live birth ratio and the body weight of sham and HIE-treated rats.
	(A) Median live birth ratios (live births/fetuses ratio [%]) are 53.8% for the HIE group [1st quartile = 38.5, 3rd quartile = 73.3] and 87.5% for the sham-operated rats [72.3, 100]. (B) The HIE group (median = 5.3 [4.8, 5.7] g) is significantly smaller than that of the sham group (6.4 [5.5, 6.8] g) at PD1. (C) The body weight of the HIE group (155 [148, 165] g) is significantly smaller than that of the sham group (185 [169, 210] g) at PD50. (D) No significant difference is observed among the body weights of each group using the Steel-Dwass multiple comparison test. Veh, 158 [149, 186] g; P4 (0.1), 155 [148, 163] g; P4 (0.01), 155 [151.5, 159.5] g; Allo, 152 [133, 194] g; MPA, 148 [143.5, 154.5] g. The body of the box plot represents the first and third quartiles of the distribution, and the median. The whiskers extend from the quartiles to the last data point within 150% of the interquartile range, with outliers beyond represented as dots.
* denotes P < 0.05 detected using the Wilcoxon rank sum test.
Abbreviations: HIE, hypoxic ischemic encephalopathy; PD1, postnatal day 1; PD50, postnatal day 50; Sham, sham-operated; Veh, vehicle-treated; P4 (0.1), 0.1 mg/body/day of progesterone-treated; P4 (0.01), 0.01 mg/body/day of progesterone-treated; Allo, allopregnanolone-treated; MPA, medroxyprogesterone 17-acetate-treated.

## Slide 4
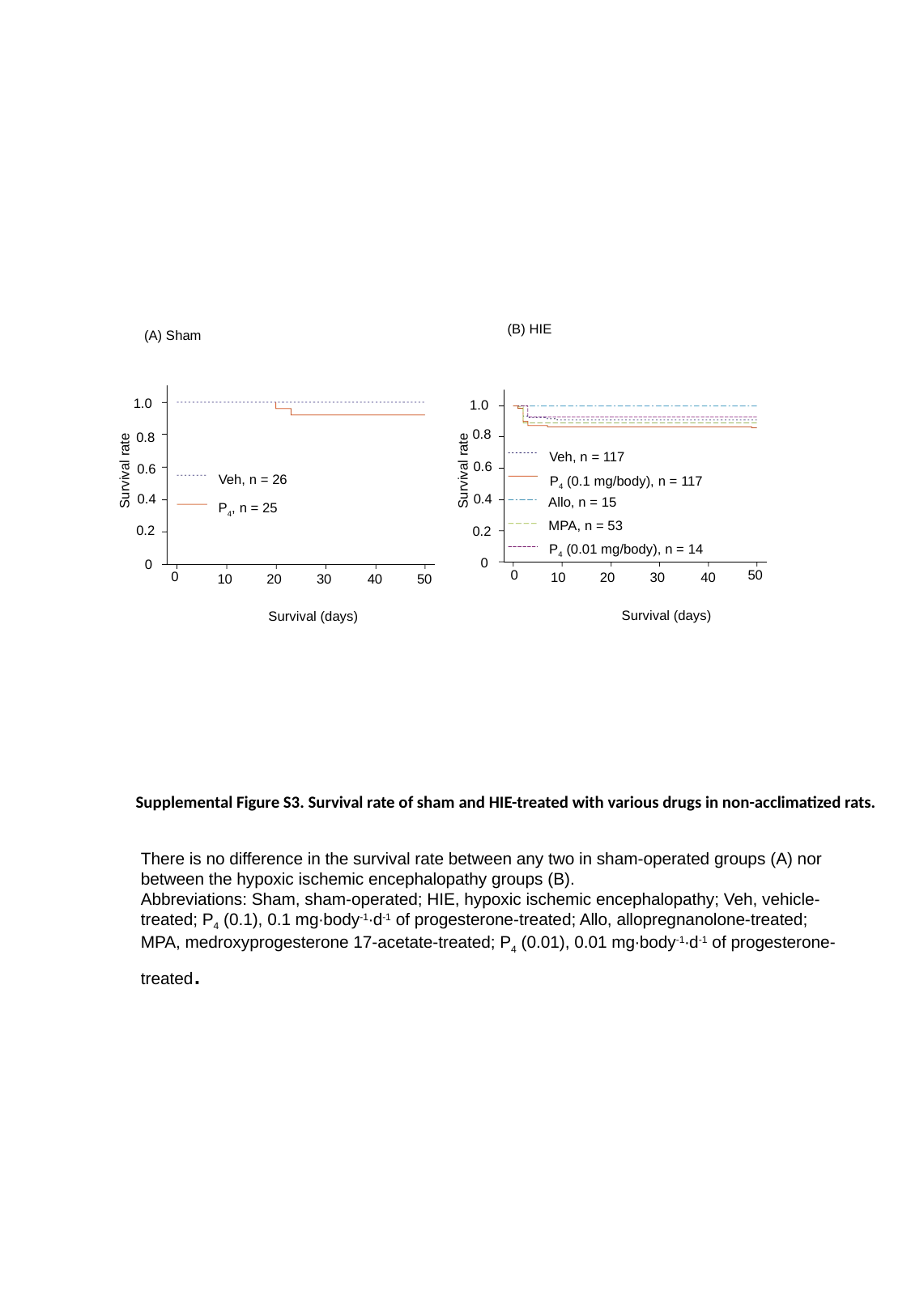

(B) HIE
 (A) Sham
1.0
Survival rate
Veh, n = 26
P4, n = 25
0
0
50
 Survival (days)
20
30
10
40
0.8
0.6
0.4
0.2
1.0
Veh, n = 117
Survival rate
P4 (0.1 mg/body), n = 117
Allo, n = 15
MPA, n = 53
P4 (0.01 mg/body), n = 14
0
0
50
 Survival (days)
0.8
0.6
0.4
0.2
20
30
10
40
Supplemental Figure S3. Survival rate of sham and HIE-treated with various drugs in non-acclimatized rats.
There is no difference in the survival rate between any two in sham-operated groups (A) nor between the hypoxic ischemic encephalopathy groups (B).
Abbreviations: Sham, sham-operated; HIE, hypoxic ischemic encephalopathy; Veh, vehicle-treated; P4 (0.1), 0.1 mg∙body-1∙d-1 of progesterone-treated; Allo, allopregnanolone-treated; MPA, medroxyprogesterone 17-acetate-treated; P4 (0.01), 0.01 mg∙body-1∙d-1 of progesterone-treated.
